# Treatment of Frey's Syndrome With “Ready to Use Liquid” Relabotulinumtoxin A (Relfydess)

**DOI:** 10.1002/lio2.70618

**Published:** 2026-10-20

**Authors:** Loris Zoller, Philipp Reddiess, Justus Müller‐Goebel, Tobias Kleinjung, Daniel Runggaldier

**Affiliations:** ^1^ Department of Otorhinolaryngology, Head and Neck Surgery University Hospital Zurich Zurich Switzerland; ^2^ University of Zurich Zurich Switzerland; ^3^ Division of Phoniatrics and Speech Pathology, Department of Otorhinolaryngology, Head and Neck Surgery University Hospital Zurich Zurich Switzerland

**Keywords:** botulinum toxin type A, Frey's syndrome, gustatory sweating, parotidectomy, Relabotulinumtoxin A, relfydess

## Abstract

**Objective:**

To describe the off‐label intradermal use of ready‐to‐use liquid relabotulinumtoxinA (Relfydess) for symptomatic Frey's syndrome after parotidectomy.

**Methods:**

A 72‐year‐old man with gustatory sweating and flushing after total parotidectomy and adjuvant radiotherapy, who had previously responded to conventional botulinum toxin A, underwent gustatory provocation, clinical mapping, and intradermal relabotulinumtoxinA injections using a 1‐cm^2^ grid. Clinical response and treatment‐related adverse events were assessed at 6 weeks and 3 months.

**Results:**

At 6 weeks and 3 months, the patient reported symptom reduction comparable to his previous treatment with conventional botulinum toxin A. At 3 months, repeat gustatory stimulation elicited no visible sweating. No treatment‐related adverse events were observed.

**Conclusion:**

In this patient, ready‐to‐use liquid relabotulinumtoxinA provided sustained symptom control for 3 months without observed adverse events. This formulation may offer a practical off‐label option for previously responsive patients, while product‐specific potency units should not be considered interchangeable.

**Level of Evidence:**

5

## Graphical abstract

We report the off‐label use of ready‐to‐use liquid relabotulinumtoxinA in a 72‐year‐old man with symptomatic Frey's syndrome after total parotidectomy and adjuvant radiotherapy. Intradermal treatment guided by clinical mapping and a 1‐cm^2^ grid achieved symptom control comparable to previous conventional botulinum toxin A at 6 weeks and 3 months, without treatment‐related adverse events.

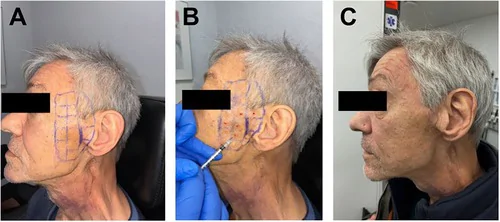

## 1 Introduction

To the best of our knowledge, this is the first reported off‐label use of ready‐to‐use RelabotulinumtoxinA (Relfydess) in a case of Frey's syndrome.

Frey's syndrome (auriculotemporal syndrome) is characterized by gustatory sweating and skin erythema in the parotid fossa and commonly occurs after parotidectomy [1, 2]. It is thought to result from aberrant regeneration of parasympathetic cholinergic fibers that reinnervate cutaneous vessels and eccrine sweat glands [1, 2]. Although objective tests may yield positive results in many patients, only a subset experiences bothersome symptoms with social or functional impact [1, 2]. In symptomatic cases, intradermal botulinum toxin type A (BoNT‐A) is a well‐established, minimally invasive treatment, often guided by clinical mapping and/or Minor's starch–iodine test [2, 3]. RelabotulinumtoxinA (Relfydess) is the first ready‐to‐use BoNT‐A formulation supplied as a liquid, thereby reducing preparation time as well as the risk of reconstitution or dilution errors. Reflydess has been evaluated in aesthetic trials and is approved in Switzerland for the cosmetic treatment of glabellar and lateral canthal lines, but very little data on clinical applications is available [4, 5].

## 2 Case Report

A 72‐year‐old patient was diagnosed with a salivary duct carcinoma of the left parotid gland (pT1 pN3b cM0). Given the extent of regional disease, he underwent total left parotidectomy combined with a radical neck dissection (levels Ib to IV). Postoperatively, due to high‐risk pathologic features (extranodal extension and perineural invasion involving the facial nerve), adjuvant radiotherapy was administered to the parotid bed and high‐risk nodal regions (levels II and V) at a total dose of 60 Gy in 30 fractions (2 Gy per fraction). Subsequently, he developed pronounced gustatory sweating and flushing involving the left preauricular skin and upper cervical region, consistent with Frey's syndrome. The diagnosis was made on clinical basis. Objective confirmation with Minor's starch–iodine test was not required, as gustatory provocation could be used to clearly delineate the extensive affected area for treatment. He initially received intradermal treatment with conventional BoNT‐A preparation (Allergan, Botox, a sterile vacuum dried botulinumtoxin, that had to be constituted in sterile saline according to standard procedures). Following gustatory provocation and clinical delineation of the sweating area, the skin was divided into a 1‐cm^2^ grid and BoNT‐A injected at 20 sites (5 IU per site; total 100 IU). The patient reported significant relief of symptoms without side effects. When symptoms recurred and the patient requested further treatment, he was offered intradermal ready‐to‐use relabotulinumtoxinA (Relfydess) after a written informed consent for off‐label usage had been obtained. After stimulation with lemon‐juice, the affected area was delineated and injected intradermally using a 1‐cm^2^ grid pattern as shown in Figure 1A,B (0.05 mL of Relfydess per cm^2^, corresponding to 5 Relfydess units per cm^2^). At 6‐week and 3‐months, the patient reported a comparable reduction in gustatory sweating and flushing compared to the prior conventional BoNT‐A treatment. After gustatory stimulation with lemon‐juice, there was no flush or sweating in the affected area as shown in Figure 1C. No treatment‐related adverse events were observed (including facial weakness, dysphagia, xerostomia, infection, or persistent injection‐site pain).

**FIGURE 1:**
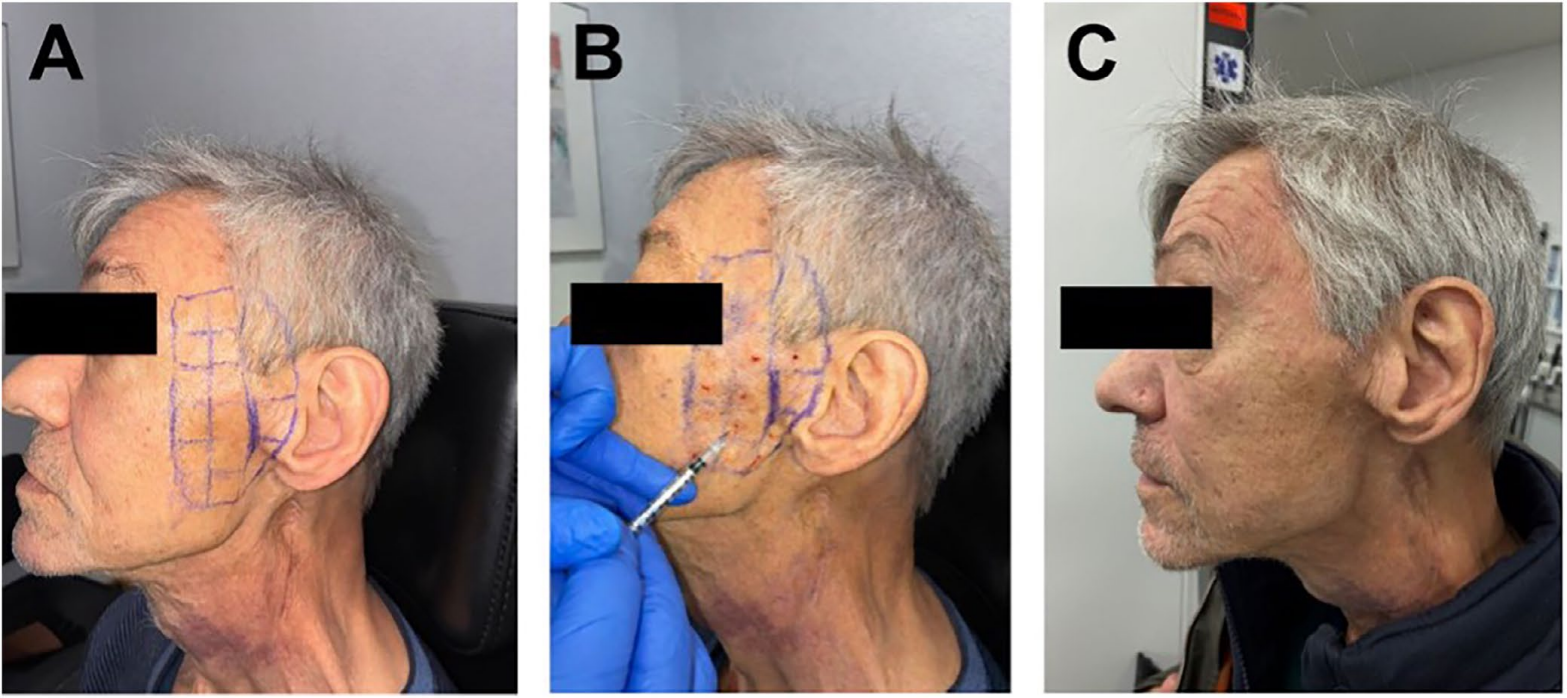
Clinical handling of RelabotulinumtoxinA/Relfydess. Clinical demonstration of gustatory sweating before and after grid‐based intradermal injection. (A) Pretreatment provoked gustatory sweating by digesting lemon juice within a mapped grid. (B) Intradermal injection performed in a grid‐based pattern across the mapped area (hand and syringe visible). (C) Follow up at 6‐weeks post‐injection after repeat provocation demonstrating no visible sweating.

## 3 Discussion

We report to the best of our knowledge and belief on the first off‐label use of ready‐to‐use RelabotulinumtoxinA (Relfydess) in a case of Frey's syndrome. This case illustrates the management of Frey's syndrome following extensive oncologic parotid surgery and adjuvant radiotherapy. While the classic presentation is gustatory sweating and skin flushing, the severity of symptoms varies greatly; objective signs of gustatory sweating are common, but clinically relevant impairments rarely occur [1, 2]. The underlying mechanism is thought to be cholinergic parasympathetic misdirection to cutaneous sweat glands and vessels after disruption of the innervation of the parotid bed [1, 2].

Multiple treatments have been described. Conservative options include reassurance, avoidance of triggers, topical antiperspirants, and topical anticholinergics (e.g., glycopyrrolate or scopolamine), but the benefit may be incomplete and side effects (skin irritation or systemic anticholinergic symptoms) may limit their use [2]. Surgical barrier techniques (e.g., interposition flaps) are primarily used for prevention at the time of parotidectomy, and therapeutic surgery is generally reserved for refractory cases [1, 2]. For symptomatic patients, intradermal BoNT‐A is commonly referrred because it directly targets cholinergic neurotransmission at eccrine glands, can be delivered on an outpatient setting, and can be repeated if symptoms reoccur [2, 3].

RelabotulinumtoxinA (Relfydess) is supplied as a ready‐to‐use liquid formulation and has been developed and evaluated for aesthetic indications, which can reduce preparation time and the risk of reconstitution or dilution errors [4]. In Switzerland, it is approved only for the treatment of glabellar and lateral canthal lines [5]. However, it is not approved for Frey's syndrome, and clinical evidence for autonomic disorders is currently lacking. In the present case, a patient with a documented response to a conventional BoNT‐A product experienced comparable relief after changing to RelabotulinumtoxinA using a mapped, grid‐based intradermal injection approach—thereby enabling the advantage of faster preparation time and eliminating the risk of reconstitution errors at the same time. However, careful symptom provocation, precise clinical mapping, and a standardized grid pattern are required to enable reproducible results when changing BoNT‐A formulations. Furthermore, explicit counseling is needed that potency units are formulation‐specific and not interchangeable, which can lead to different results [4].

Limitations of that report include the case report of a single patient, reliance on patient‐reported outcomes, and the relatively short follow‐up period of 3 months. Prospective studies are needed to determine dose–response relationships, duration of effect, comparative safety and convenience (including cost‐effectiveness) of ready‐to‐use BoNT‐A formulations in the clinical setting.

## 4 Conclusion

Off‐label ready‐to‐use liquid RelabotulinumtoxinA/Relfydess can provide symptom relief comparable to a conventional BoNT‐A preparation in Frey's syndrome when delivered with careful clinical mapping and grid‐based injections, without observed adverse effects—enabling advantages such as faster preparation time and elimination of reconstitution errors. Further prospective studies are required to assess the value of RelabotulinumtoxinA/Relfydess in broader clinical applications.

## Funding

The authors have nothing to report.

## Disclosure

During the preparation of this manuscript, the authors used OpenAI ChatGPT to assist with drafting, language editing, and structural refinement. The AI tool was not used for data generation, data analysis, interpretation of clinical findings, or clinical decision‐making. All AI‐assisted content was critically reviewed, verified against the clinical data and cited source literature, and edited by the authors, who take full responsibility for the final content.

## Ethics Statement

Formal ethical approval was waived by the local Ethics Committee (Kantonale Ethikkommission, Zurich, Switzerland) due to the retrospective design of the study, its basis on individual cases, and the fact that all procedures were conducted as part of routine clinical care.

## Conflicts of Interest

The authors declare no conflicts of interest related to this work.

## Data Availability

The data that support the findings of this study are available on request from the corresponding author. The data are not publicly available due to privacy or ethical restrictions.
